# Non-additive strong gene interactions cause striking differences in organ pathology and cytokine response in Leishmaniasis

**DOI:** 10.3389/fimmu.2025.1579257

**Published:** 2025-10-14

**Authors:** Yahya Sohrabi, Tatyana Kobets, Valeriya Volkova, Eliška Javorková, Imtissal Krayem, Alena Zajícová, Helena Havelková, Milena Svobodová, Vladimír Holáň, Peter Demant, Marie Lipoldová

**Affiliations:** ^1^ Department of Medical Genetics, Third Faculty of Medicine, Charles University, Prague, Czechia; ^2^ Laboratory of Molecular and Cellular Immunology, Institute of Molecular Genetics, Czech Academy of Sciences, Prague, Czechia; ^3^ Department of Cardiology I, Coronary, Peripheral Vascular Disease and Heart Failure, University Hospital Münster, University of Münster, Münster, Germany; ^4^ Department of Toxicology and Molecular Epidemiology, Institute of Experimental Medicine of the Czech Academy of Sciences, Prague, Czechia; ^5^ Laboratory of Leukocyte Signalling, Institute of Molecular Genetics, Czech Academy of Sciences, Prague, Czechia; ^6^ Department of Parasitology, Faculty of Science, Charles University, Prague, Czechia; ^7^ Department of Molecular and Cellular Biology, Roswell Park Comprehensive Cancer Center, Buffalo, NY, United States; ^8^ Laboratory of Signal Transduction, Institute of Molecular Genetics, Czech Academy of Sciences, Prague, Czechia

**Keywords:** leishmaniasis, host-pathogen interaction, mouse model, novel genetic mechanisms of disease susceptibility and resistance, CD11b + Gr1 + cells, asymptomatic leishmaniasis

## Abstract

The mouse strain O20 is highly resistant to parasite *Leishmania major*. O20 mice differed from all resistant strains tested until now, as they harbored parasites in their organs, but upon exposure to soluble *Leishmania* antigen (SLA) their splenocytes did not respond by cytokine production and their macrophages did not produce NO, suggesting a novel mechanism of resistance. Another resistant strain C57BL/10 (B10) harbors similar numbers of parasites as O20 in its organs and its splenocytes respond to SLA by production of IFNγ, but not IL-4. They also produce IL-2, IL-6, IL-10 and IL-17. Macrophages respond to SLA by NO production. Strain B10.O20 was derived from a cross of these two resistant strains. B10 provided 96.4% of its genome and O20 contributed 3.6% of its genome. Unexpectedly, this very limited difference between the two strains resulted in the very large phenotypic effects. B10.O20 was susceptible to *L. major*, as it exhibited large skin lesions, high parasite numbers in skin and lymph nodes, and a massive spleen infiltration by CD11b^+^CD193^+^ and CD11b^+^Gr1^+^ cells. Thus, a small percentage of genes of the resistant strain O20 in the genome of the second resistant strain B10 resulted in high susceptibility to *L. major*. After stimulation with SLA, splenocytes of B10.O20 produced significantly higher levels of all Th1, Th2 and Th17 cytokines than both its parental strains B10 and O20. This suggested a chronic inflammation with imbalance of several arms of immune response. In summary, the responses of strains B10.O20 and O20 to *L. major* revealed novel disease phenotypes that have not been observed previously in mice but they were seen in several clinical studies of human leishmaniasis. The studies of heterogeneity of defensive strategies of mouse strains may guide development of effective antileishmanial therapies or vaccine development and it could serve as a basis for investigation of asymptomatic responses to other infectious diseases.

## Graphical Abstract

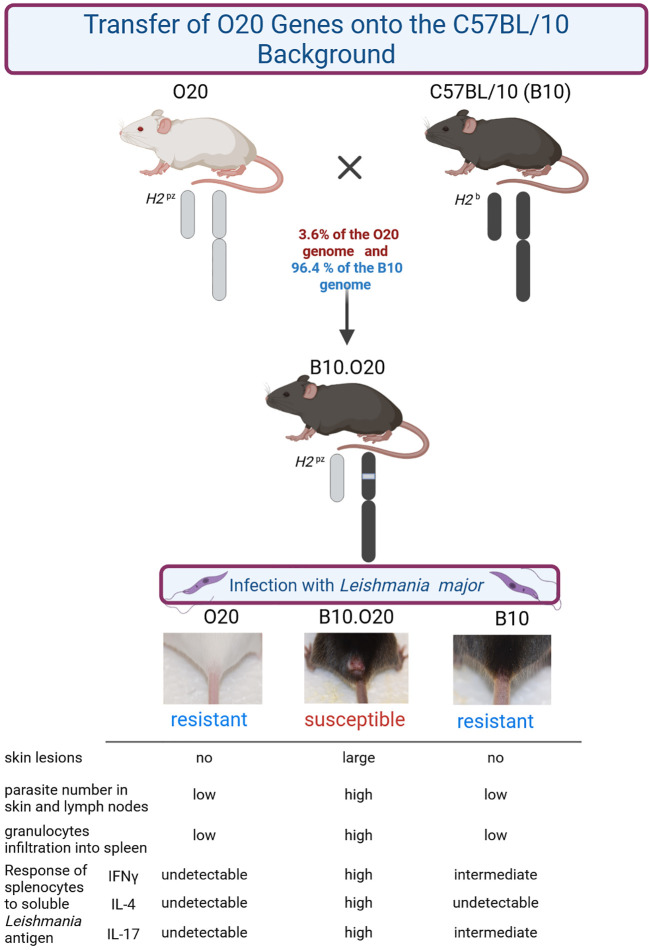

## Introduction

More than 1 billion people living in areas endemic for leishmaniasis are at risk of infection by *Leishmania* – an obligatory intracellular protozoan parasite (Kinetoplastida) of vertebrates, transmitted by female phlebotomine sandflies (1–4). The principal vertebrate host target cell is the macrophage, where parasites proliferate, but *Leishmania* parasites can also invade many other cell types (5–7). Three main clinical syndromes of leishmaniasis were described: the cutaneous form of the disease in dermis, which can be localized or diffuse; mucocutaneous leishmaniasis in the mucosa, and the visceral leishmaniasis that results from the spread of infection to the spleen and liver (8, 9). Currently, there is no human vaccine to prevent leishmaniasis and drugs used for treatment have serious side effects (4, 10, 11).

In addition to people with clinical signs of the leishmaniasis, there are at least 120 million people with asymptomatic infection (12). While they do not manifest any symptoms of the disease, their serological, PCR (polymerase chain reaction) or leishmanin skin tests are positive (13). In some areas these subclinical infections represent 80-90% of those infected [reviewed in (14)]. These clinically asymptomatic individuals can serve as reservoirs for transmission of parasites (15, 16). The persistent parasites also pose a risk to asymptomatic individuals, as they can be reactivated in immunocompromised host (17, 18). The resistance or susceptibility to the infection and manifestations of the disease depend on *Leishmania* species, and on genetic and non-genetic factors such as age, sex, and social and environmental influences (2, 19–25).

It is difficult to control these multiple factors in analyses of human infection, therefore many aspects of infection were analyzed in animal experiments. Especially the mouse studies with large numbers of genetically identical individuals kept in controlled conditions helped to establish many elements of mechanical and genetic control of the disease (19, 26–30). The most extensively studied mouse model has been infection by *Leishmania major.* This parasite causes cutaneous lesions in humans, but induces both cutaneous and systemic pathology in mouse. Most information about response to infection was obtained using the two paradigmatic strains: susceptible BALB/c and resistant C57BL/6N (B6) (23, 26).

Following inoculation of *L. major*, susceptible BALB/c mice exhibit uncontrolled growth of the parasite in the primary site of infection, development of large necrotic lesions and dissemination of parasites beyond the local draining lymph node to spleen, liver, bone marrow, and other cutaneous sites (31, 32), whereas resistant B6 mice develop neither cutaneous nor systemic disease (31).

Several early studies suggested that resistance or susceptibility to infection caused by *L. major* was controlled by the behavior of different subpopulations of helper T cells (33, 34), because the amount of IL-4 produced by T helper 2 (Th2) cells appeared to correlate with progression of leishmaniasis, whereas activation of IFNγ-producing Th1 lymphocytes, which promotes the production of nitric oxide in phagocytes at the site of infection (35), has been associated with resolution of the disease. Subsequent studies revealed that both T and non-T compartments contributed to determination of the susceptibility or resistance (36) and identified additional cells and cytokines involved to the susceptibility (32, 37). They include dendritic cells (38, 39), neutrophils (40, 41), keratinocytes (42), myeloid-derived suppressor cells (43), and microRNA (44).

Multiple regulatory factors revealed in mechanistic studies have been supported by genetic analyses that established a network-like organization of the numerous functionally diverse genes influencing susceptibility to leishmaniasis (19, 23, 27, 45–49).

Despite these insights into the immune response against *Leishmania* parasites, there are indication that additional responses required for protection against the disease remain to be defined (10, 50). This is supported by the fact that there is currently no safe and effective vaccine that would prevent any form of human leishmaniasis (4, 10, 11, 50, 51).

This might be partly caused by the fact that the majority of analyses had been performed only on a few mouse strains (26, 52), which often originated from common ancestors (53). This does not reflect the multiple genotypes existing in the human population.

In order to develop an additional model for analysis of susceptibility to *L. major*, we tested response to *L. major* in strains O20/A (O20), C57BL/10Sn (B10), and compared it to that of C57BL/10-*H2^pz^* (B10.O20). The strain B10.O20 is a *H2* congenic strain on the B10 background (N8), which carries *H2^pz^* haplotype derived from the strain O20 (54). Because the inbreeding of the strain B10.O20 started at the eighth backcross generation, approximately 3.6% of its genome, including *H2* region, are derived from the strain O20 (55). Analysis of susceptibility to *L. major* in the strains O20, B10 and B10.O20 revealed that the combination of genes of two resistant strains, O20 and B10, present in the strain B10.O20 caused its susceptibility. Here we show that this susceptibility of B10.O20 mice is associated with novel immunological and pathological characteristics of leishmaniasis of this strain. We also observed that the strain O20 exhibits a paradoxical combination of high resistance to infection and a defective response to SLA (soluble *Leishmania* antigen) previously unreported in mice. In order to facilitate the understanding of these novel phenotypes, we give a detailed description of the immunological parameters of uninfected and infected O20, B10 and B10.O20 mice.

## Materials and methods

### Mice

The first set of experiments comprised: 147 (71 infected and 76 uninfected) female mice of strains O20/A (abbrev. O20) total 57 (23 infected and 34 uninfected), B10.O20/R164/Dem (abbrev. B10.O20) (The Jackson Laboratory Strain 38154/MMRRC: 069936) total 51 (24 infected and 27 uninfected) and C57BL/10Sn (abbrev. B10) total 39 (24 infected and 15 uninfected) that were tested in 5 independent experiments. Age of mice was 12 to 28 weeks (mean 17.8 weeks, median 16.1 weeks) at the time of infection. 3 mice died during experiments and one mouse, which had injury (was bitten), was excluded from calculation.

The second set of experiments: 35 (18 infected and 17 uninfected) female mice of strains BALB/c (6 infected and 6 uninfected), STS (6 infected and 5 uninfected) and O20 (6 infected and 6 uninfected) were tested in 2 independent experiments. Age of mice was 9 to 15 weeks (mean 12.4 weeks, median 13 weeks) at the time of infection.

Mice were euthanized by cervical dislocation after 8 weeks of infection. Blood, skin, spleen, liver and inguinal lymph nodes were collected for later analysis.

### Ethical statement

All experimental protocols utilized in this study comply with the Czech Government Requirements under the Policy of Animal Protection Law (No.246/1992) and with the regulations of the Ministry of Agriculture of the Czech Republic (No.207/2004), which are in agreement with all relevant European Union guidelines for work with animals. The study was approved by the Institutional Animal Care Committee of the Institute of Molecular Genetics of the Czech Academy of Sciences and by Departmental Expert Committee for the Approval of Projects of Experiments on Animals of the Czech Academy of Sciences (permission Nr. 93/2015).

### Parasite

*Leishmania major* LV 561 (MHOM/IL/67/LRCL 137 JERICHO II) was maintained in rump lesions of BALB/c females. Amastigotes were transformed to promastigotes using SNB-9 medium (56). 10^7^ promastigotes from the passage number 2 cultivated for six days were inoculated in 50 μl sterile saline s.c. into mouse rump (57).

### Disease phenotype

The size of the skin lesions was measured every second week using the Profi LCD Electronic Digital Caliper Messschieber Schieblehre Messer (Shenzhen Xtension Technology Co., Ltd. Guangdong, China), which has accuracy 0.02 mm.

### Quantification of parasite load by PCR-ELISA

Parasite load was measured in frozen skin, lymph nodes, spleen, and liver samples using PCR-ELISA according to the previously published protocol (58). Briefly, total DNA was isolated using a TRI reagent (Molecular Research Center, Cincinnati, USA) standard procedure (https://www.mrcgene.com/dna-isolation) or a modified proteinase K procedure (58). For PCR, two primers (digoxigenin-labeled F 5′-ATT TTA CAC CAA CCC CCA GTT-3′ and biotin-labeled R 5′-GTG GGG GAG GGG CGT TCT-3′ (VBC Genomics Biosciences Research, Austria) were used for amplification of the 120-bp conservative region of the kinetoplast minicircle of *Leishmania* parasite, and 50 ng of extracted DNA was used per each PCR reaction. For a positive control, 20 ng of *L. major* DNA per reaction was amplified as a highest concentration of standard. A 30-cycle PCR reaction was used for quantification of parasites in lymph nodes, and 35 cycles for skin, spleen, and liver. Under these conditions the amount of PCR product is linearly proportional to the number of parasites (58). PCR product was measured by the modified ELISA (Pharmingen, San Diego, USA). Concentration of *Leishmania* DNA was determined using the ELISA Reader Tecan and the curve fitter program KIM-E (Schoeller Pharma, Prague, Czech Republic) with least squares-based linear regression analysis.

### Preparation of soluble *Leishmania* antigen

*L. major* LV 561 (MHOM/IL/67/LRC-L137 JERICHO II) promastigotes were harvested from RPMI 1640 medium supplemented with 10% inactivated fetal calf serum (Sigma-Aldrich, USA), 63.7 μg/ml penicillin (Sigma-Aldrich, USA), and 100 μg/ml streptomycin (Sigma-Aldrich, USA), washed 3 times with PBS (pH 7.2) and used for preparation of soluble *Leishmania* antigen (SLA) as previously described (59). Briefly, 100 μl of protease inhibitor cocktail enzyme (Sigma, St. Louis, MO, USA) was added to 1 × 10^9^ promastigotes, the parasites were freeze-thawed 10 times followed by sonication at 4°C with two 20-sec blasts. The parasite suspension was then centrifuged at 30,000× g for 20 min at 4°C, the supernatant was collected and re-centrifuged at 100,000× g for 4 h at 4°C, and the resulting supernatant was filtered through a 0.22 μm MILLEX^®^ GP Syringe Filter Unit (Millipore, Carrigtwohill, Co., Cork, Ireland). Protein concentration of SLA was determined using Lowry method, and the SLA preparation was aliquoted and stored at −20°C until used. In first and second sets of experiments (see the above section Mice) were used different batches of SLA.

### Stimulation of splenocytes or macrophages by SLA

Single cell suspensions of spleen cells were prepared in RPMI 1640 medium (Sigma Corp., St. Louis, MO) containing 10% of FCS (Sigma), antibiotics (100 U/ml of penicillin, 100 μg/ml of streptomycin), 10 mM HEPES buffer, and 5 x 10^-5^ 2-mercaptoethanol. Spleen cells at a concentration 0.6 x 10^6^ cells/ml were incubated in 48-well tissue culture plates (Corning Inc., Corning, NY) in a final volume of 1 ml of complete RPMI 1640 medium unstimulated or stimulated with 6 µg/ml of soluble *Leishmania* antigen (SLA). The supernatants were harvested after a 24-h (IL-2 detection), 48-h (IFN-γ detection) or 72-h (IL-4, IL-6, IL-10 and IL-17 detection) incubation at 37°C in an atmosphere of 5% CO_2_.

To produce and measure IL-1β, IL-12 and NO, peritoneal exudate cells (PEC) containing 20-30% of macrophages (60) obtained by washing the peritoneal cavity of tested mice were adjusted to a concentration of 1 × 10^6^ cells/ml and were stimulated with 6 µg/ml of SLA. The supernatants were harvested after 48-h incubation for detection of NO or after 72 h for quantification of IL-1β and IL-12.

### Cytokine detection and quantification by ELISA

The production of IL-1β, IL-2, IL-4, IL-6, IL-10, IL-12, IL-17 and IFNγ was quantified by ELISA. The pairs of cytokine-specific capture and detection monoclonal antibodies (mAb) purchased from R&D Systems, Inc. (Minneapolis, MN) were used for the detection of IL-2 (mAb clones JES6-5H4 and JES6-1A12), IL-6 (mAb clones MP5-20F3 and MP5-32C11) and IFNγ (mAb clones R4-6A2 and XM6-1.2). IL-1β, IL-4, IL-10, IL-12 and IL-17 were measured using ELISA kits (DuoSet ELISA Development System: IL-4 (kit DY404), IL-10 (DY417), IL-17 (DY421), IL-1β (DY401), IL-12 (DY499)) purchased from R & D Systems (Minneapolis, MN), following the instructions of the manufacturer. The reaction was quantified by spectrophotometry using a Sunrise Remote ELISA Reader (Gröding, Austria).

### Nitric oxide measurement

Briefly, 100 μl of the cell culture supernatant was incubated with 50 μl of 1% sulfanilamide (in 3% H_3_PO_4_) and 50 μl of 0.3% N-1-napthyletylenediamine dihydrochloride (in 3% H_3_PO_4_) at room temperature for 5 min. Nitrite was quantified by spectrophotometry at 550 nm using sodium nitrite as a standard (61).

### Flow cytometry characterization of spleens from infected and uninfected mice

Single-cell suspensions prepared from spleens of infected and uninfected mice were washed in PBS containing 0.5% BSA and incubated for 30 min on ice with the following anti-mouse mAb (all purchased from BioLegend, San Diego, CA): allophycocyanine (APC)-labeled anti-CD11b (clone M1/70), fluorescein isothiocyanate (FITC)-labeled anti-Ly6G/Ly-6C (Gr-1) (clone RB6-8C5), FITC-labeled anti-CD19 (clone 6D5), Alexa Fluor 647-labeled anti-CD22 (clone OX-97), phycoerythrin-labeled anti-CD193 (clone J073E5), APC-labeled anti-CD3 (clone 17A2), FITC-labeled anti-CD4 (clone GK1.5), PE-labeled anti-CD8 (clone 53-6.7), APC-labeled anti-CD80 (clone 16-10A1), FITC-labeled anti-CD206 (clone C068C2), PE-labeled anti-F4/80 (clone BM8), PE-labeled anti-CD14 (clone Sa 14-2). All samples were also incubated with Pacific Blue-labeled anti-TER-119 (clone TER-119) in order to exclude erythroid cells. Dead cells were stained using Hoechst 33258 fluorescent dye (Invitrogen, Carlsbad, CA) added to the samples 10 min before flow cytometry analysis. Data were collected using an LSRII cytometer (BD Biosciences, Franklin Lakes, NJ) and analyzed using FlowJo software (Tree Star, Ashland, OR). Fifty thousand events from each sample were measured.

### Evaluation of histopathological changes in spleen

Organs were fixed in 4% formaldehyde and embedded in paraffin. Histological changes were evaluated in hematoxylin-eosin (H&E) stained 2 μm sections under a light microscope.

### Statistical analysis

Differences between strains O20, B10.O20 and B10, and between infected and uninfected mice within tested strains were analyzed by Mann-Whitney test using the program Statistica for Windows 12.0 (StatSoft, Inc., Tulsa, Oklahoma, USA).

## Results

### Combination of genes of two resistant strains results in a susceptible strain

**Figure 1:**
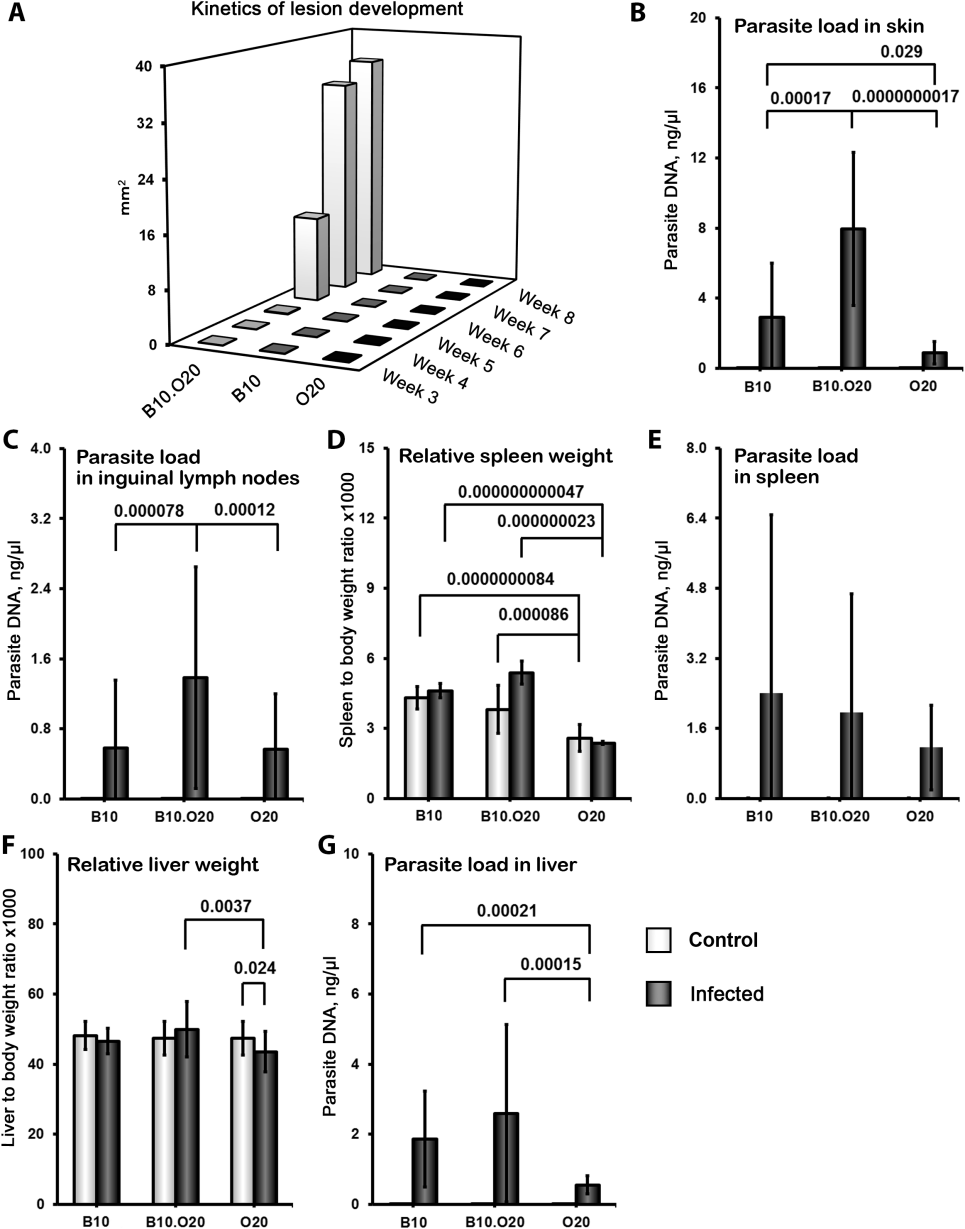
Differences in skin lesions, relative spleen and liver weight and parasite numbers in organs after *L. major* infection. Female mice of strains O20 (23 infected, 28 uninfected), B10 (23 infected, 11 uninfected) and B10.O20 (21 infected, 23 uninfected) were compared. Animals were subcutaneously inoculated with 10^7^ promastigotes of *L. major.* Control, uninfected mice were kept in the same animal facility. Both groups were killed after 8 weeks of infection. The data show the medians (**A** - skin lesions) and the means ± SD **(B–G)** from five independent experiments.

**Figure 2:**
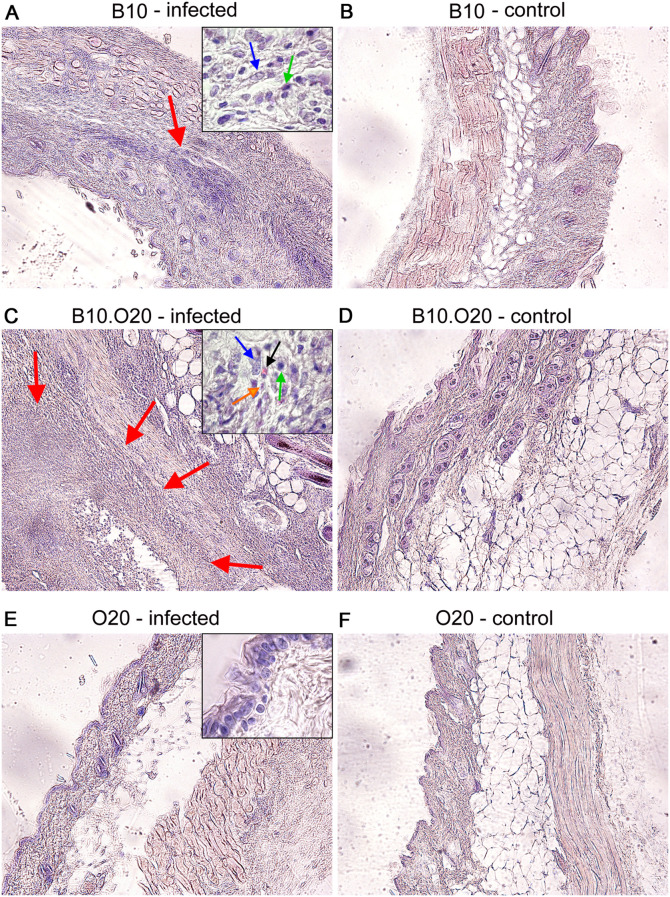
Comparison of the strain influence on architecture of infected skin in O20, B10, and B10.O20 mice. Photos show hematoxylin-eosin (H&E) stained skin sections of infected **(A, C, E)** and uninfected **(B, D, F)** female mice at x100 magnification. Red arrows point areas of epithelioid granulomas. Infected mice of susceptible B10.O20 strain developed extensive granulomas with massive infiltration of various immune cells and parasites. In the high magnification inserts, blue, green and black arrows point macrophages, lymphocytes and eosinophils, respectively. Orange arrow shows presence of amastigotes in skin of B10.O20.

Previously, *H2^pz^* haplotype of the *Leishmania*-resistant strain O20 was transferred on the background of another resistant strain C57BL/10Sn-*H2^b^* (B10) to create a strain C57BL/10-*H2^pz^* (B10.O20) (62). We have tested these three strains, B10, O20 and B10.O20, for susceptibility to *L. major* and found that the combination of genomes of the two resistant strains B10 and O20, gave rise to the strain B10.O20 (**Figure 1**), which was susceptible and had larger skin lesions (**Figure 1A**), higher number of parasites in skin (**Figure 1B**) and in inguinal lymph nodes (**Figure 1C**) than either resistant parental strain. The presence of high numbers of amastigotes in the skin of B10.O20 was also confirmed by anti-*Leishmania* staining (**Supplementary Figure 1**). B10.O20 also developed extensive epithelioid granulomas in the skin (**Figure 2C**), whereas B10 had only isolated granulomas (**Figure 2A**) and O20 mice did not develop any epithelioid granulomas (**Figure 2E**). Skins of uninfected control mice are shown in **Figures 2B, D, F**.

In addition, considerable pathological changes were observed also in spleens of B10.O20 mice, but not in B10 and O20 (see the next section).

### Spleens of susceptible strain B10.O20 exhibit upon infection extensive disorganization and high myeloid-derived cells infiltration

**Figure 3:**
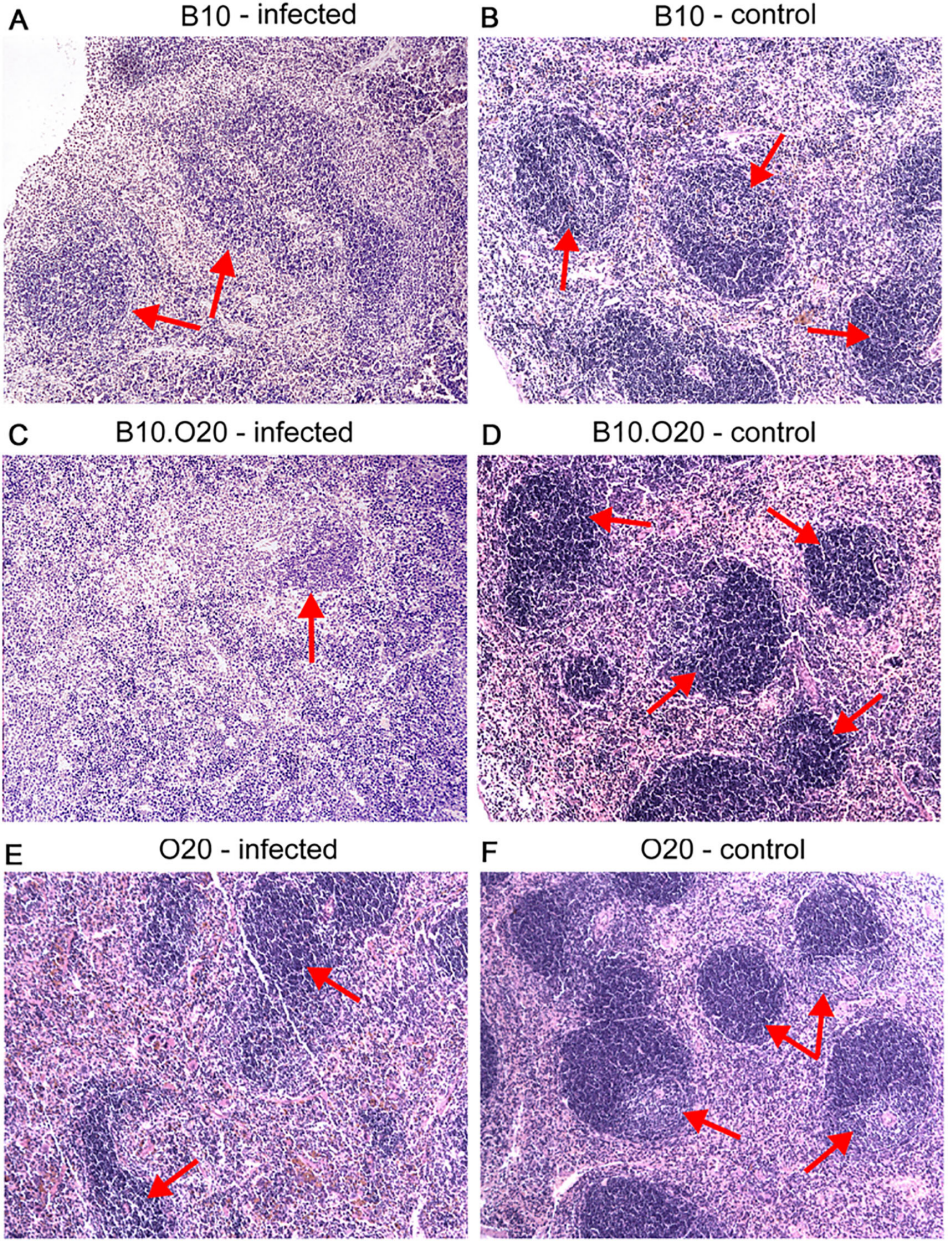
Microarchitecture of the spleen. Photos show microstructure of spleen of infected **(A, C, E)** and uninfected **(B, D, F)** female mice at x100 magnification, hematoxylin–eosin stained cuts. Arrows point areas of preserved white pulp. Infected mice exhibit signs of white pulp activation, especially notable in the susceptible B10.O20 strain **(C)**.

**Figure 4:**
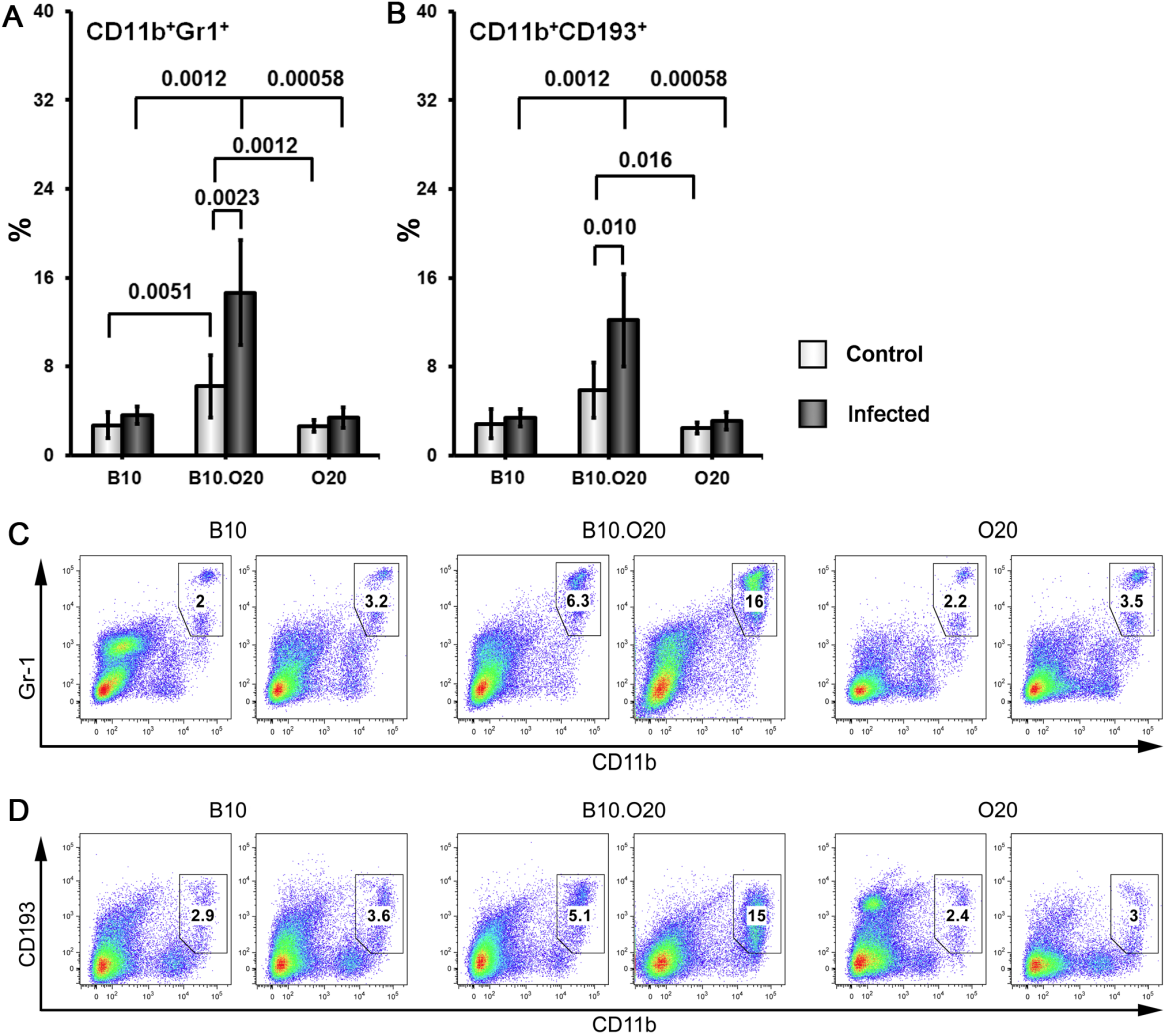
Flow cytometric analysis of freshly obtained mouse spleen cells. Female mice of strains O20 (7 infected, 6 uninfected), B10 (6 infected, 5 uninfected) and B10.O20 (7 infected, 7 uninfected) were compared. Frequencies of CD11b+Gr1+ cells **(A, C)** and CD11b+CD193+ cells **(B, D)** are shown. Dead cells were stained using Hoechst 33258 fluorescent dye added to the samples 10 min before flow cytometry analysis. Cells were stained with allophycocyanine (APC)-labeled anti-CD11b (clone M1/70), fluorescein isothiocyanate (FITC)-labeled anti-Ly6G/Ly-6C (Gr-1) (clone RB6-8C5) and phycoerythrin-labeled anti-CD193 (clone J073E5) antibodies as described in Materials and Methods. Selected cell populations were gated on single cell events after exclusion of cell debris and dead cells. Figure shows means ± SD from 4 independent experiments and a representative FACS experiment.

**Figure 5:**
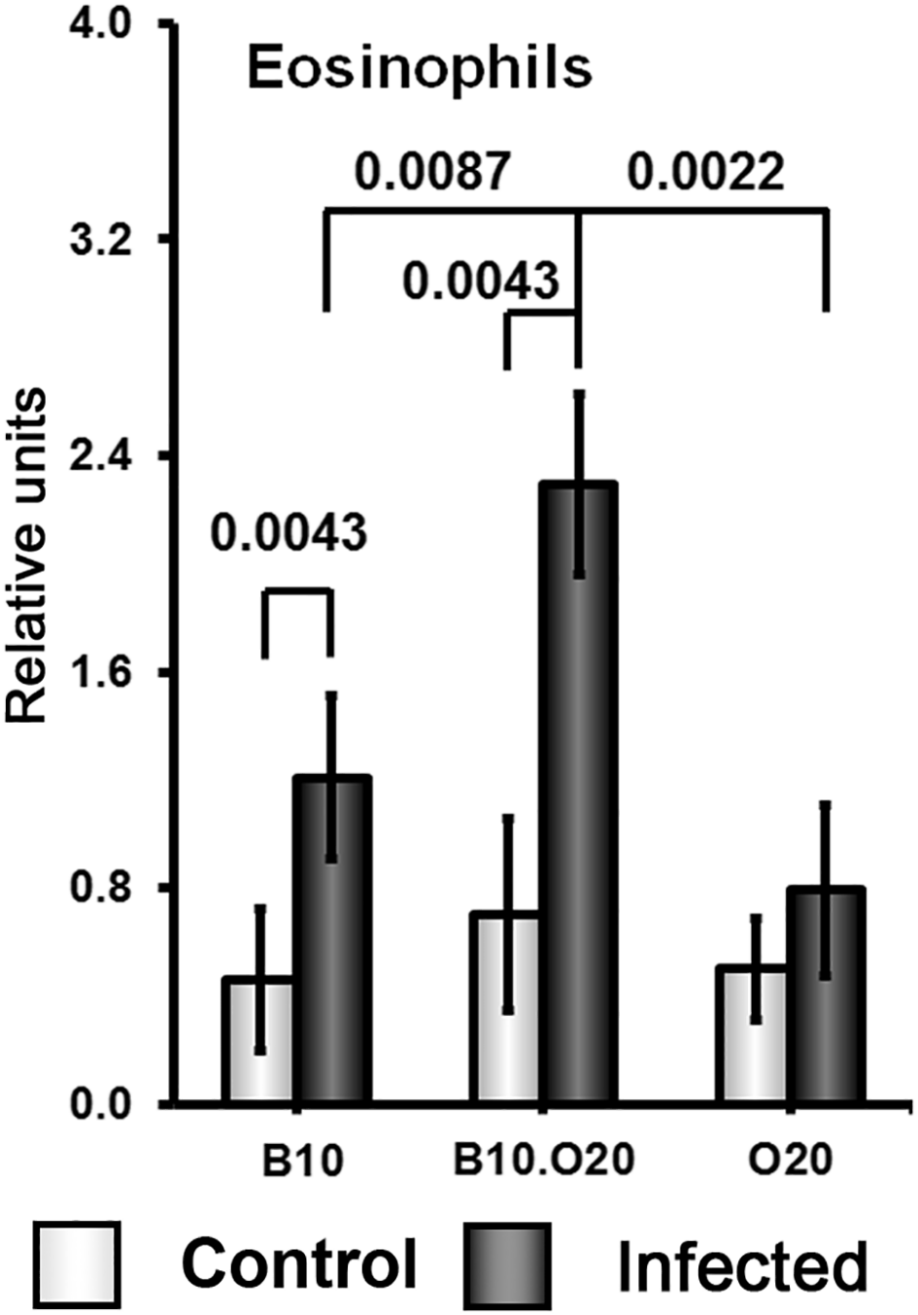
Eosinophil infiltration in spleens of infected and uninfected mice. Female mice of strains O20 (6 infected, 6 uninfected), B10 (6 infected, 6 uninfected) and B10.O20 (6 infected, 5 uninfected) were compared. The data show the means ± SD from two independent experiments. The analysis was performed on H&E sections.

Strain O20 has a lower relative spleen weight than both B10 and B10.O20 prior infection (**Figure 1D**). None of the three strains exhibited significant increase of relative spleen weight after infection (**Figure 1D**) and tested strains also did not significantly differ in parasite load in spleen (**Figure 1E**). **Figure 3** shows microarchitecture of spleens of uninfected (**Figures 3B, D, F**) and infected (**Figures 3A, C, E**) mice. Spleens of B10.O20 were extensively disorganized (**Figure 3C**), having lost the distinction between red and white pulp. They had also a higher frequency of CD11b^+^Gr1^+^ (**Figures 4A, C**) and CD11b^+^CD193^+^ (**Figures 4B, D**) subpopulations than both B10 (*P* = 0.0012, *P* = 0.0012, respectively) and O20 (*P* = 0.00058, *P* = 0.00058, respectively). The frequency of CD11b^+^Gr1^+^ subpopulation in the uninfected strain B10.O20 was higher than in both parental strains, and the *L. major* infection led to dramatic increase of these cells (**Figures 4A, C**). Similarly, uninfected B10.O20 had higher frequency of CD11^+^CD193^+^ (eosinophils) subpopulation than the strain O20; infection led to increase of CD11^+^CD193^+^ in B10.O20, but not in B10 and O20, which led to the increase of the difference between B10.O20 and its parental strains (**Figures 4B, C**). Higher infiltration of eosinophils in spleens of B10.O20 mice was confirmed also by observation of high numbers of eosinophils in histological examination (**Figure 5**).

**Figure 6:**
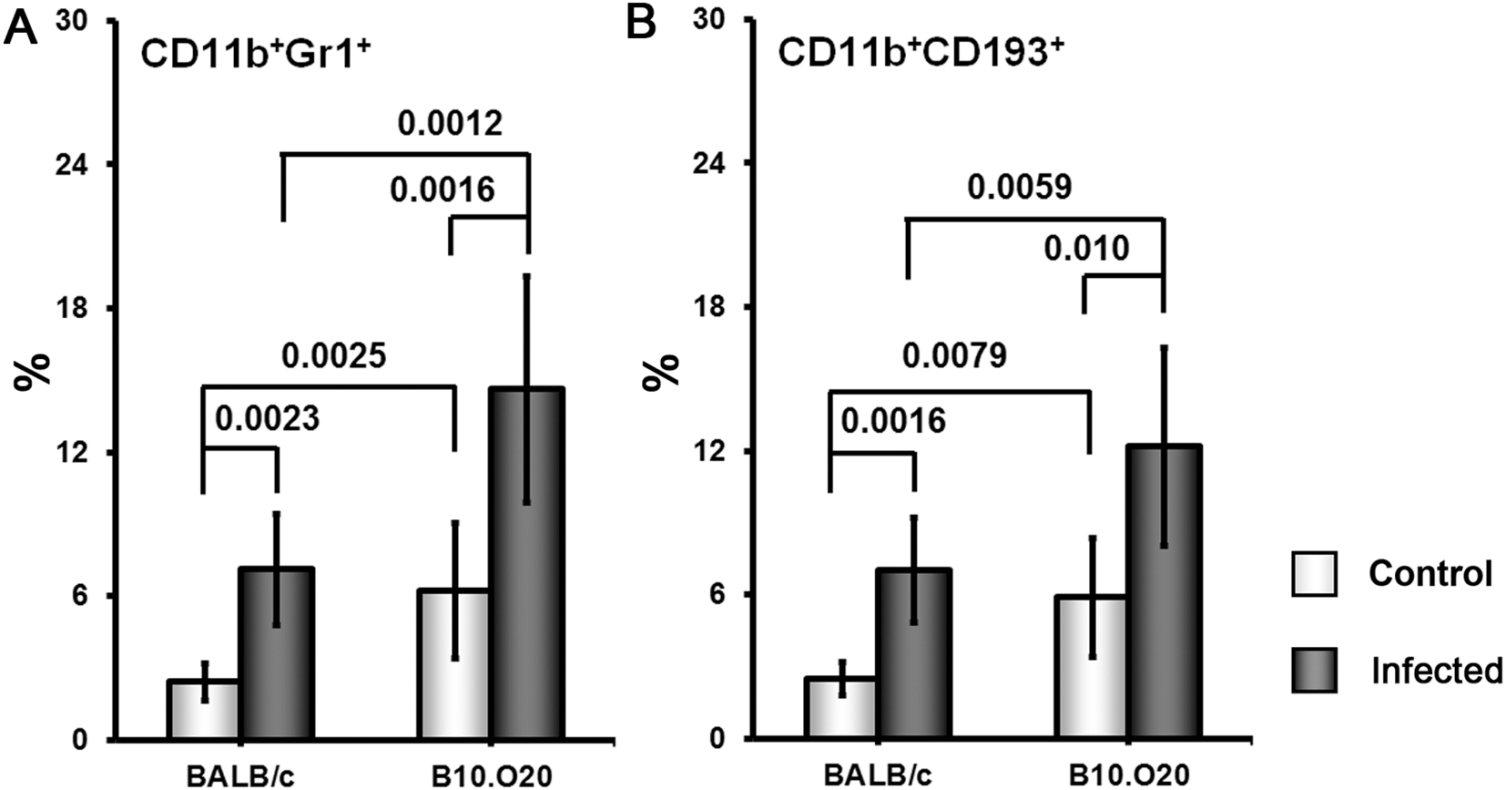
Comparison of frequency of CD11b^+^Gr1^+^ **(A)** and CD11b^+^CD193^+^ **(B)** cells in spleens of the strains B10.O20 and BALB/c. Female mice of strains BALB/c (8 infected, 5 uninfected) and B10.O20 (7 infected, 7 uninfected) were analyzed by flow cytometry. Dead cells were stained using Hoechst 33258 fluorescent dye added to the samples 10 min before flow cytometry analysis. Cells were stained with allophycocyanine (APC)-labeled anti-CD11b (clone M1/70), fluorescein isothiocyanate (FITC)-labeled anti-Ly6G/Ly-6C (Gr-1) (clone RB6-8C5) and phycoerythrin-labeled anti-CD193 (clone J073E5) antibodies as described in Materials and Methods. Selected cell populations were gated on single cell events after exclusion of cell debris and dead cells. Figure shows means ± SD from 2 independent experiments and a representative FACS experiment.

Because of the striking high frequency of CD11b^+^Gr1^+^ and CD11^+^CD193^+^ subpopulations in the strain B10.O20, we decided to compare it with the most intensively studied susceptible strain BALB/c (**Figure 6**). We found that uninfected B10.O20 mice have higher frequency of both these subpopulations than uninfected BALB/c and this difference increased after *L. major* infection.

Spleens of infected B10.O20 mice exhibited also higher frequency of monocyte derived CD14^+^ (**Supplementary Figure 2A**) and F4/80^+^ (**Supplementary Figure 2B**) subpopulations and a lower frequency of T cell helper CD3^+^CD4^+^ (**Supplementary Figure 2C**), T cell cytotoxic CD3^+^CD8^+^ (**Supplementary Figure 2D**) and B lymphocyte CD19^+^CD22^+^ (**Supplementary Figure 2E**) subpopulations than both B10 and O20. B10.O20 spleens display a higher frequency of cells carrying T cell stimulation receptor CD86^+^ (**Supplementary Figure 2F**) than O20 and lower than B10. However, these differences were less pronounced than differences in CD11b^+^Gr1^+^ and CD11b^+^CD193^+^ subpopulations, and except CD19^+^CD22^+^ subpopulation were already present in uninfected mice (**Supplementary Figure 2**).

Infected mice did not show a significant increase of relative liver weight after infection (**Figure 1F**). The parasite load in liver of B10.O20 exhibited “B10-like” responses. Both B10.O20 (*P* = 0.00015) and B10 (*P* = 0.00021) exhibit higher parasite load in liver than O20 (**Figure 1G**). B10.O20 exhibited “B10-like” responses also in other liver parameters. Both B10 and B10.O20 had larger number and size of granulomas than O20 (**Supplementary Figures 3A, B**), and more macrophages (**Supplementary Figure 3C**), eosinophils (**Supplementary Figure 3D**), neutrophils (**Supplementary Figure 3E**) and lymphocytes (**Supplementary Figure 3F**) in granulomas. However, none of the histological liver parameters (except neutrophil content in granulomas, which was higher in infected than in uninfected B10.O20 mice (*P* = 0.030)) changed after infection and they mirrored differences in granulomas numbers, size and composition already present in uninfected mice (**Supplementary Figure 3**).

### Soluble *Leishmania* antigen stimulated high cytokine production in the susceptible strain B10.O20 and intermediate in resistant strain B10, which contrasted with low production in the resistant strain O20

**Figure 7:**
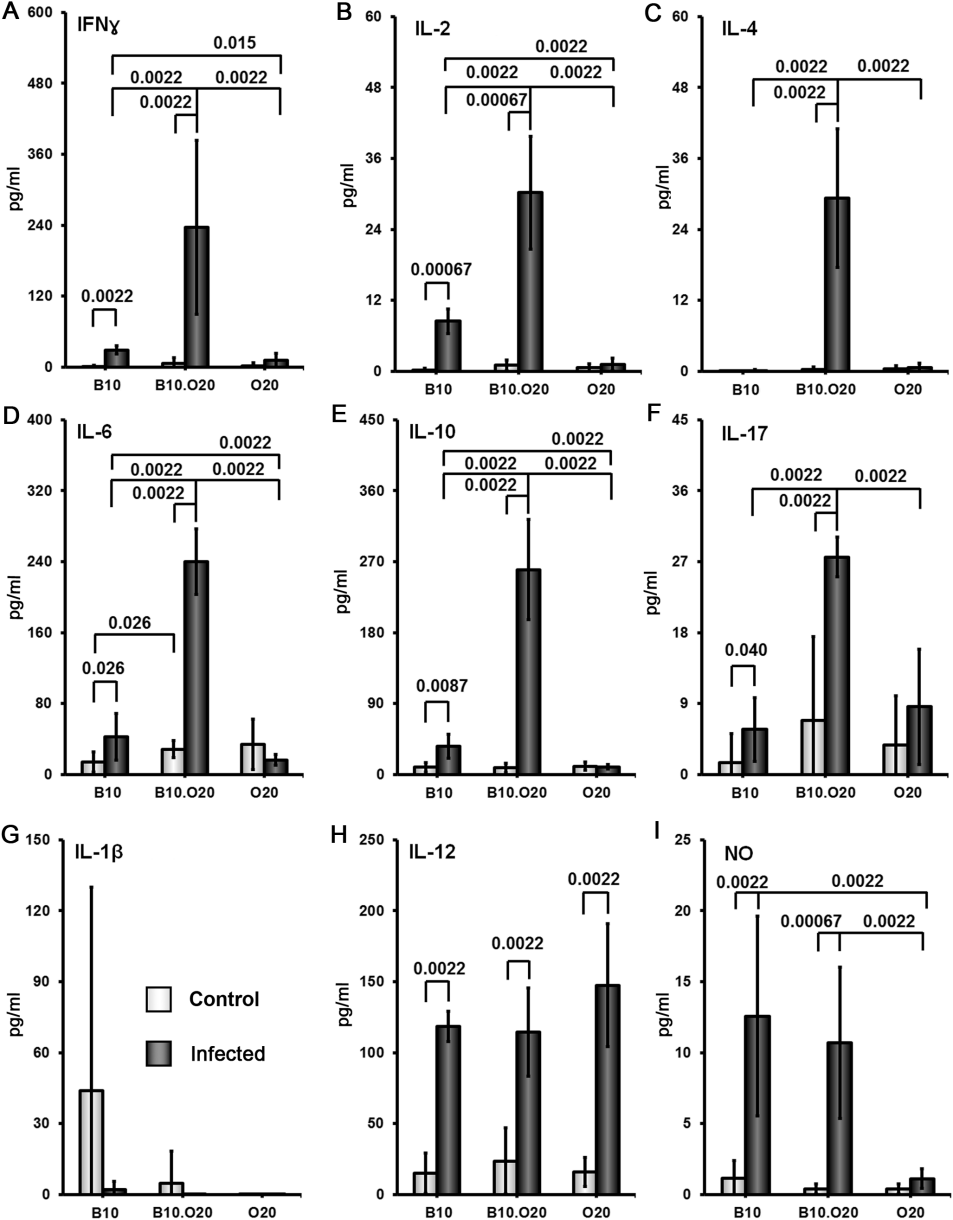
Comparison of cytokine and NO response to the soluble *Leishmania* antigen (SLA). Female mice of strains O20, B10 and B10.O20 were compared. At least 6 uninfected and 6 infected mice from each strain were analyzed. Production of IFNγ **(A)**, IL-2 **(B)**, IL-4 **(C)**, IL-6 **(D)**, IL-10 **(E)** and IL-17 **(F)** by splenocytes and production of IL-1β **(G)**, IL-12 **(H)** and NO **(I)** by peritoneal exudate cells was determined. The data show the means ± SD from 5 independent experiments.

Splenocytes of uninfected mice of all strains produced low or undetectable levels of tested cytokines after stimulation with SLA. Infection of the strain B10.O20 resulted in 15-350× increase of splenocytes’ production of IFNγ (**Figure 7A**) IL-2 (**Figure 7B**), IL-4 (**Figure 7C**), IL-6 (**Figure 7D**), IL-10 (**Figure 7E**) and IL-17 (**Figure 7F**). Cells of the strain B10 also exhibit increased production of IFNγ IL-2, IL-6, IL-10 and IL-17, but not to the extent of the strain B10.O20 (**Figure 7**). The splenocytes of the resistant strain O20 practically do not respond to SLA by production of cytokines. The production of IFNγ, IL-4 and IL-17 by O20 is undetectable and production of IL-2, IL-10 and IL-6 by this strain was very low.

After SLA stimulation peritoneal macrophages of O20 mice do not produce NO, in contrast to B10 and B10.O20 macrophages (**Figure 7I**), but there was no difference in production of IL-1β (**Figure 7G**) and IL-12 (**Figure 7H**) by SLA-stimulated macrophages among the tested strains. Macrophages of the resistant strain B10 and susceptible strain B10.O20 did not differ in production of NO. Surprisingly, macrophages of the resistant strain O20 produce IL-12, but not IL-1β and NO (**Figures 7G–I**).

**Figure 8:**
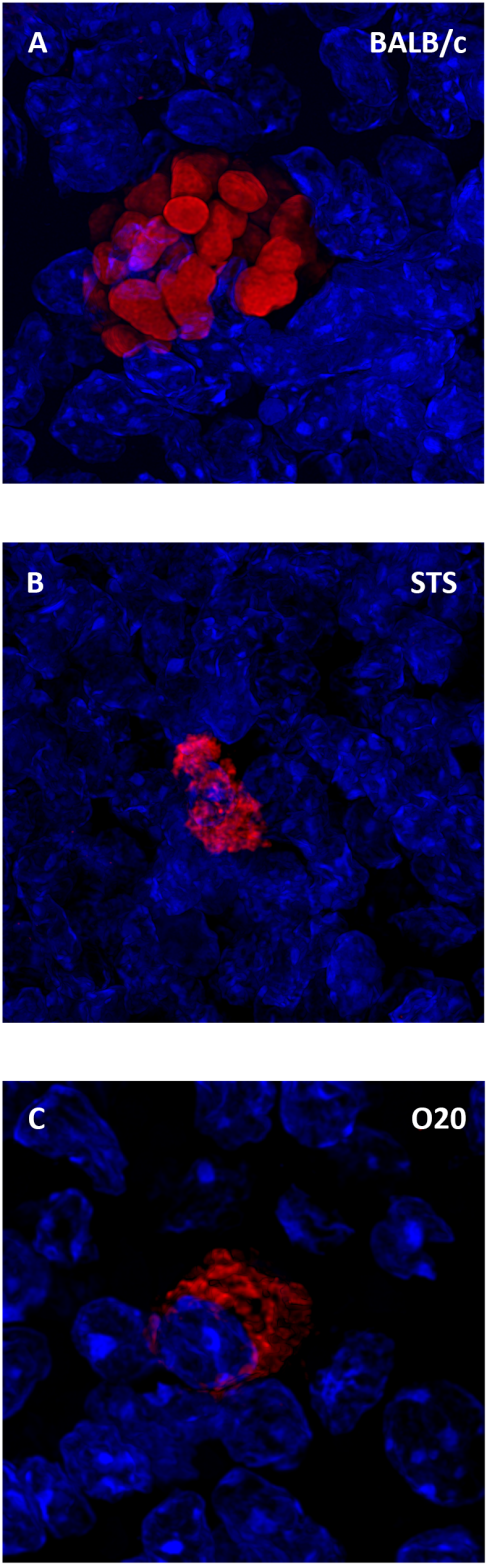
*Leishmania major* parasites inside the spleen. Slices of spleen tissue of females of BALB/c **(A)**, STS **(B)** and O20 **(C)** mice were stained with the anti-*Leishmania* lipophosphoglycan mouse monoclonal antibody (cat. no. CLP003A, Cedarlane, Hornby, Canada) and TRITC labelled IgM (115-025-020, Jackson ImmunoResearch, West Grove, PA) all diluted 1:500. Nuclei of the cells were stained with Bisbenzimide H33258 (Sigma-Aldrich, St. Louis, MO) 10 mg per 1 ml diluted 1:1000. Images were captured with confocal microscope Leica TCS SP8 objective HC PL APO 63×/1.40 OIL.

**Figure 9:**
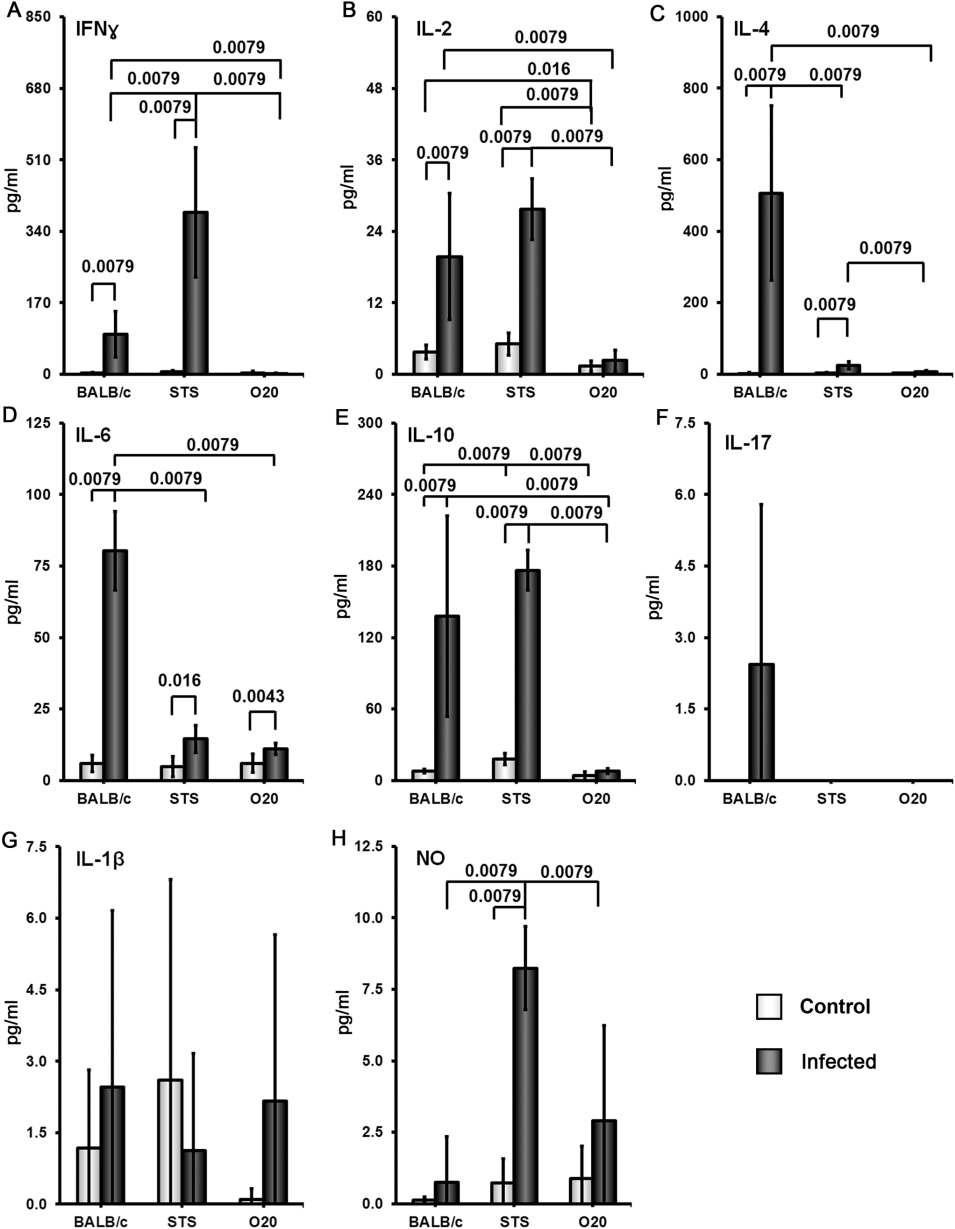
Comparison of cytokine and NO response to the soluble *Leishmania* antigen (SLA).Female mice of the strains O20, BALB/c and STS were compared. At least 5 uninfected and 6 infected mice from each strain were analyzed. Production of IFNγ **(A)**, IL-2 **(B)**, IL-4 **(C)**, IL-6 **(D)**, IL-10 **(E)** and IL-17 **(F)** by splenocytes and production of IL-1β **(G)** and NO **(H)** by peritoneal exudate cellswas estimated. The data show the means ± SD from 2 independent experiments.

Surprising lack of cytokine response to SLA by the strain O20 prompted us to compare it with another *Leishmania* resistant strain STS and the widely studied *Leishmania* susceptible strain BALB/c. All these strains contain parasites in their spleens (**Figure 8**). Similarly as in previous tests, splenocytes of uninfected BALB/c, STS and O20 mice produce low or undetectable levels of tested cytokines after stimulation with SLA. Infection of BALB/c and STS mice resulted in 10-500× increase of production of IFNγ (**Figure 9A**) IL-2 (**Figure 9B**), IL-4 (**Figure 9C**), IL-6 (**Figure 9D**) and IL-10 (**Figure 9E**) by their splenocytes, whereas strain O20 splenocytes did not produce any of these cytokines except IL-6 (**Figure 9D**). None of the tested strains showed significant increase in production of IL-17 (**Figure 9F**) by splenocytes and increase of IL-1β by peritoneal macrophages (**Figure 9G**). The increase of NO by peritoneal macrophages was observed only in the strain STS (**Figure 9H**).

In summary, we have analyzed two susceptible strains, B10.O20 and BALB/c, and three resistant strains: B10, O20 and STS. These five strains exhibited five different patterns of splenocytes SLA response. B10.O20: high levels of IFNγ, IL-2, IL-4, IL-6, IL-10 and IL-17; BALB/c: high levels of IL-2, IL-4, IL-6, IL-10 and intermediate levels of IFNγ and IL-17; STS: high levels of IFNγ, IL-2 and IL-10, low to intermediate level of IL-4, intermediate level of IL-6 and undetectable level of IL-17, B10: intermediate level of IFNγ, IL-2, IL-6, IL-10 and IL-17 and undetectable level of IL-4; O20 did not contain detectable concentration of tested cytokines with the exception of IL-6.

## Discussion

We show that the combination of genes of two resistant strains, O20 and B10, which is present in the strain B10.O20, unexpectedly results in its susceptibility and extremely high numbers of CD11b^+^Gr1^+^ cells in B10.O20 mice before and even more after infection. In addition, we observed in strains O20 and B10.O20 several previously unreported phenotypes, namely a high resistance to infection in O20 mice accompanied by non-responsiveness to the SLA antigen. In view of the potential conceptual importance of the responses to *L. major* in these strains, we report in detail these immunological and immunopathological phenotypes. The parameters that appear to be most closely associated with response to *L. major* are given in the main body of the paper, those not altered after infection are given in detail in **Supplementary Figures**.

### Strong combinatorial genetic influence on response to *Leishmania* infection

We have observed that combination of genes of two resistant strains gave rise to a susceptible strain. There might be several causes of this phenomenon: a) epistasis, b) combination of effects of minor “susceptibility” alleles of resistant strains, c) combination of a) and b).

a) *Epistasis*. Influence of genetic background on phenotypic effects was discovered in studies of many genes disrupted by targeting: for example *Igf1r* (insulin-like growth factor I receptor) (63), *Egfr* (epidermal growth factor receptor) (64), *Fcgr2b* (Fc receptor, IgG, low affinity Iib) (65), as well as on naturally mutated genes *Map3k14* (mitogen-activated protein kinase kinase kinase 14) (66) and polymorphic haplotypes *H2* ^b^, *H2* ^d^ (67), and *H2* ^d^, *H2* ^k^ (68). These effects are attributable to modifier genes, which act in combination with the causative gene, differ on different genetic backgrounds and were identified only in few cases (68).

We have previously identified loci/genes that function differently on B10 background in control of frequency of CD11b+Gr1+ subpopulation in spleens of uninfected B10.O20 (55). This parameter is controlled by three loci *Mydc1* (myeloid-derived cells 1), *Mydc2* and *Mydc3* on chromosomes 1, 15 and 17, respectively. We identified potential candidate genes for *Mydc1* (*Smap1*) and for *Mydc3* (*Vps5*2, *Tnxb* and *Rab44*). *Mydc1* exhibits single gene effect, whereas the influence of *Mydc2* and *Mydc3* was observed only in their interaction. In all three loci, the alleles controlling higher frequency of CD11b+Gr1+ cells were of O20 origin (55). Thus, the transfer of O20 alleles on B10 background led to increase of frequency CD11b+Gr1+ cells.

b) *A limited number of non-overlapping loci with susceptible alleles in O20 and B10*. These two sets of “susceptibility” alleles that became by chance accumulated in B10.O20 strain and their combined effect prevails over “resistant” alleles of B10 and O20. These “susceptibility” alleles can work additively or exhibit genetic epistasis.

### Novel *L. major* response phenotypes in O20 and B10.O20 mice

O20 and B10.O20 mice exhibit resistant and susceptible phenotypes, respectively, that have not been observed before.

The strain O20 does not develop skin lesions (**Figure 1A**) and it is more resistant to *L. major* than the widely used resistant strains B6 and 129, which develop localized transient skin lesions that heal spontaneously (69–71), and it is also more resistant than the strain STS, which develops none, or only small localized lesions (45, 57). O20 is also more resistant than the strain B10, as described in detail in Results (**Figures 1B**, **2A**). However, the mechanisms of resistance of O20 are different. Similarly as the B10 and B10.O20, O20 does not exhibit splenomegaly or hepatomegaly and does not differ from these strains in parasite load in spleen (**Figures 1D–F**), but it has more preserved white pulp (**Figures 3A, C, E**). Surprisingly, spleen cells of the infected O20 mice practically do not respond to SLA by cytokine production (**Figures 7A–F**), but its peritoneal macrophages respond to SLA by IL-12 production (**Figure 7H**). This cytokine response differs from other resistant strains: B6, B10 and 129 mice respond to SLA by high production of IFNγ and low production of IL-4 (69–71 and this paper). However, macrophages of O20 do not respond to SLA by production of NO (**Figure 7I**). NO induction is more difficult to induce in humans than in most tested rodents (72), thus the strain O20 is more “human-like” in this trait. As mentioned above, the resistant strain B10 responds to SLA with increase of production of IFNγ, but it also exhibits increase of IL-10 and IL-17 that were described to induce immunopathology (32, 73); however, increase of levels of these cytokines was less expressed than increase of IFNγ (**Figure 7**). Response of the resistant strain STS is also characterized by high and low production of IFNγ (**Figure 9A**) and IL-4 (**Figure 9C**), respectively; similarly as B10, it also responds by increase of IL-10 level (**Figure 9E**). The dichotomy in IFNγ response to SLA in the strain O20, and the strains B6, B10, 129 and STS resembles dichotomy observed in human responses to *L. donovani* antigens (74). Thus, analysis of more mouse strains might better model the heterogeneity of human responses to leishmaniasis.

The strain B10.O20 is susceptible, but to a lesser degree than the strain BALB/c. In the same experimental design, BALB/c mice suffer from approximately twice as large skin lesions as B10.O20 and develop extensive splenomegaly and hepatomegaly (57). Splenocytes of infected susceptible B10.O20 mice produce Th1, Th2 and Th17 cytokines in response to SLA. They have increased production of Th1 cytokine IFNγ which is associated with resolution of the disease (33), as well as increase of Th2 cytokines IL-4 and IL-10, and Th17 cytokine IL-17 (**Figures 7A–F**), that were described to correlate with aggravation of disease (32, 33, 73). This mixed cytokine response is similar to responses observed in multiple studies of human cutaneous (75, 76) and visceral (74, 77, 78) leishmaniasis, where patients exhibit both Th1 and Th2 responses.

Another unexpected feature of response of B10.O20 mice to *L. major* infection is an exceptionally high increase of CD11b^+^Gr1^+^ and CD11b^+^CD193^+^ subpopulations. The frequencies of these subpopulations were even higher in B10.O20 than in the prototype susceptible BALB/c mice (**Figure 6**). The myeloid-derived CD11b^+^Gr1^+^ subpopulation has been found to play role in cancer, autoimmune diseases, traumatic stress and transplantation [reviewed in (79)], as well as in infectious diseases, including leishmaniasis. Gr1^+^ monocytes from B6. but not BALB/c mice have been found to kill *L. major* parasites in the early stages of infection (80). Their role in the chronic disease has to be elucidated. We have also found increased eosinophilic infiltration in spleens of the infected B10.O20 mice (**Figure 5**). The future analysis of the phenomena described in this subsection needs to comprise also time-course studies, which are beyond the scope of the present paper.

The tested strains differed prior to infection in relative spleen weight (**Figure 1D**), frequencies in cell subpopulations in the spleen (**Figure 4**; **Supplementary Figure 2**) and in liver (**Supplementary Figure 3**). The heritable differences in gene expression (81) and metabolism (82) present prior to infection may contribute to differential susceptibility to *Bordetella pertussis* and *L. donovani*, respectively. Future studies leading to identification of genes controlling baseline parameters and genes determining susceptibility to *L. major* and their comparison will elucidate the role of baseline differences observed in the present study on response to the infection.

### Asymptomatic response

Encounter of host with pathogen can lead to no entrance or clearance of pathogen, to asymptomatic infection when host mounts a response strong enough to control pathogen, and to a symptomatic disease (83). Asymptomatic infections by viruses, bacteria and parasites are widespread. Data about the proportion of asymptomatic individuals from the first years of SARS-Cov19 epidemics varies from 1.2% in Wuhan, China (84) to 17.9% on the board of the Diamond Princess cruise ship (85). Asymptomatic norovirus prevalence was estimated to be about 7% (86). Kendall and coworkers indicated that 30% of people diagnosed through community-based testing were completely asymptomatic for *Mycobacterium tuberculosis* (87). 37.5% of *Plasmodium falciparum* infections and 18.5% of *P. vivax* infections in Brazil were asymptomatic when microscopy was used for diagnosis (88). Infection with *Toxoplasma gondii* in developed countries is asymptomatic in 80% of newly infected patients (89).

The outcome of an interplay between pathogen and host depends on many factors such as pathogen density and properties, host immunity, underlying medical conditions, as well as on age, immune and hormonal status, sex, nutrition (90, 91), and genetic background of the host (23, 92–94). Mouse experiments in controlled conditions allow to study an influence of these individual factors. In this paper, we show in mouse experiments that two different genotypes (O20 and STS) lead to asymptomatic infection by different mechanisms.

## Conclusion

The present report demonstrates the benefits of using more mouse strains to analyze disease susceptibility in order to better reflect the diverse immunological characteristics of the human population. We have observed strong effect of small percentage of genes of the resistant strain O20 on the genetic background of another resistant strain B10 in control of susceptibility to *L. major*. This is accompanied by novel phenotypes of susceptible and resistant strains exhibiting heterogeneity of defensive strategies, which resemble certain human disease phenotypes. Analysis of these novel phenotypes can help to develop new therapies and vaccines designed to treat different genotypes. Moreover, these studies could serve as a basis for the investigation of asymptomatic responses to leishmaniasis, but also to other infectious diseases.

## Data Availability

The original contributions presented in the study are included in the article/**Supplementary Material**. Further inquiries can be directed to the corresponding author.
