# Supplementary material for: Non-additive strong gene interactions cause striking differences in organ pathology and cytokine response in Leishmaniasis
Source: Front Immunol. 2025 Oct 14;16:1579257. doi: 10.3389/fimmu.2025.1579257 (PMC12558852; doi:10.3389/fimmu.2025.1579257)

SUPPLEMENTARY MATERIALS

Non-additive strong gene interactions cause striking differences in organ pathology and cytokine response in leishmaniasis

Yahya Sohrabi[1,2,3], Tatyana Kobets[2], Valeriya Volkova[2], Eliška Javorková[4], Imtissal Krayem[2,5], Alena Zajícová[4], Helena Havelková[2], Milena Svobodová[6], Vladimír Holáň[4], Peter Demant[7], Marie Lipoldová[1,2,8*]

[1]Department of Medical Genetics, Third Faculty of Medicine, Charles University, Prague, Czech Republic; [2]Laboratory of Molecular and Cellular Immunology, Institute of Molecular Genetics, Czech Academy of Sciences, Prague, Czech Republic; [3]Department of Cardiology I, Coronary, Peripheral Vascular Disease and Heart Failure, University Hospital Münster, Münster, Germany; [4]Department of Toxicology and Molecular Epidemiology, Institute of Experimental Medicine of the Czech Academy of Sciences, Prague, Czech Republic; [5]Laboratory of Leukocyte Signalling, Institute of Molecular Genetics, Czech Academy of Sciences, Prague, Czech Republic; [6]Department of Parasitology, Faculty of Science, Charles University, Prague, Czech Republic; [7]Department of Molecular and Cellular Biology, Roswell Park Comprehensive Cancer Center, Buffalo, NY, United States, [8]Laboratory of Signal Transduction, Institute of Molecular Genetics, Czech Academy of Sciences, Prague, Czech Republic

*Correspondence: marie.lipoldova@lf3.cuni.cz; lipoldova@img.cas.cz

SUPPLEMENTARY FIGURE 1. ***Leishmania major* parasites inside the skin.** Slices of skin tissue of infected females of B10.O20 and O20 mice were stained with the anti-*Leishmania* lipophosphoglycan mouse monoclonal antibody (cat. no. CLP003A, Cedarlane, Hornby, Canada) and TRITC labelled IgM (115-025-020, Jackson ImmunoResearch, West Grove, PA) all diluted 1 : 500. Nuclei of the cells were stained with bisBenzimide H33258 (Sigma-Aldrich, St. Louis, MO) 10 mg per 1 ml diluted 1:1000. Images were captured with confocal microscope Leica TCS SP8 objective HC PL APO 63×/1.40 OIL.

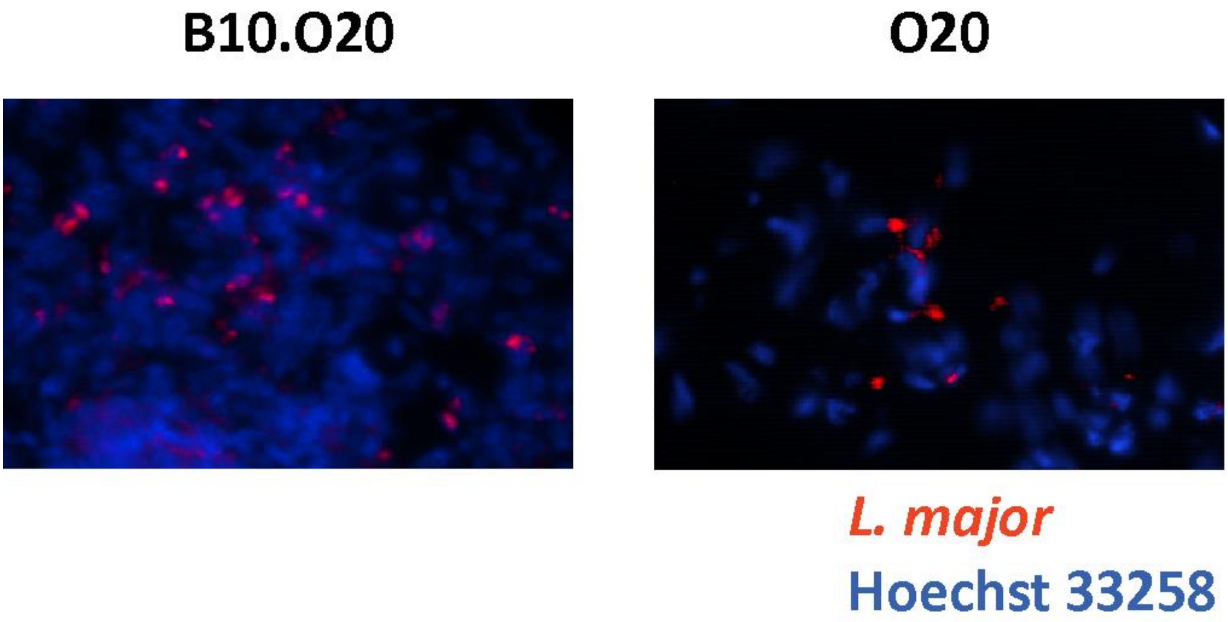

SUPPLEMENTARY FIGURE 2. Flow cytometric analysis of freshly obtained mouse spleen cells and peritoneal macrophages. Female mice of strains O20 (7 infected, 6 uninfected), B10 (6 infected, 5 uninfected) and B10.O20 (7 infected, 7 uninfected) were compared. Dead cells were stained using Hoechst 33258 fluorescent dye added to the samples 10 min before flow cytometry analysis. Cells were stained with PE-labeled anti-CD14 (clone Sa 14-2), PE-labeled anti-F4/80 (clone BM8), APC-labeled anti-CD3 (clone 17A2), FITC-labeled anti-CD4 (clone GK1.5), PE-labeled anti-CD8 (clone 53-6.7), FITC-labeled anti-CD19 (clone 6D5), Alexa Fluor 647-labeled anti-CD22 (clone OX-97) as described in Materials and Methods. Selected cell populations were gated on single cell events after exclusion of cell debris and dead cells. Figure shows means ± SD from 4 independent experiments.

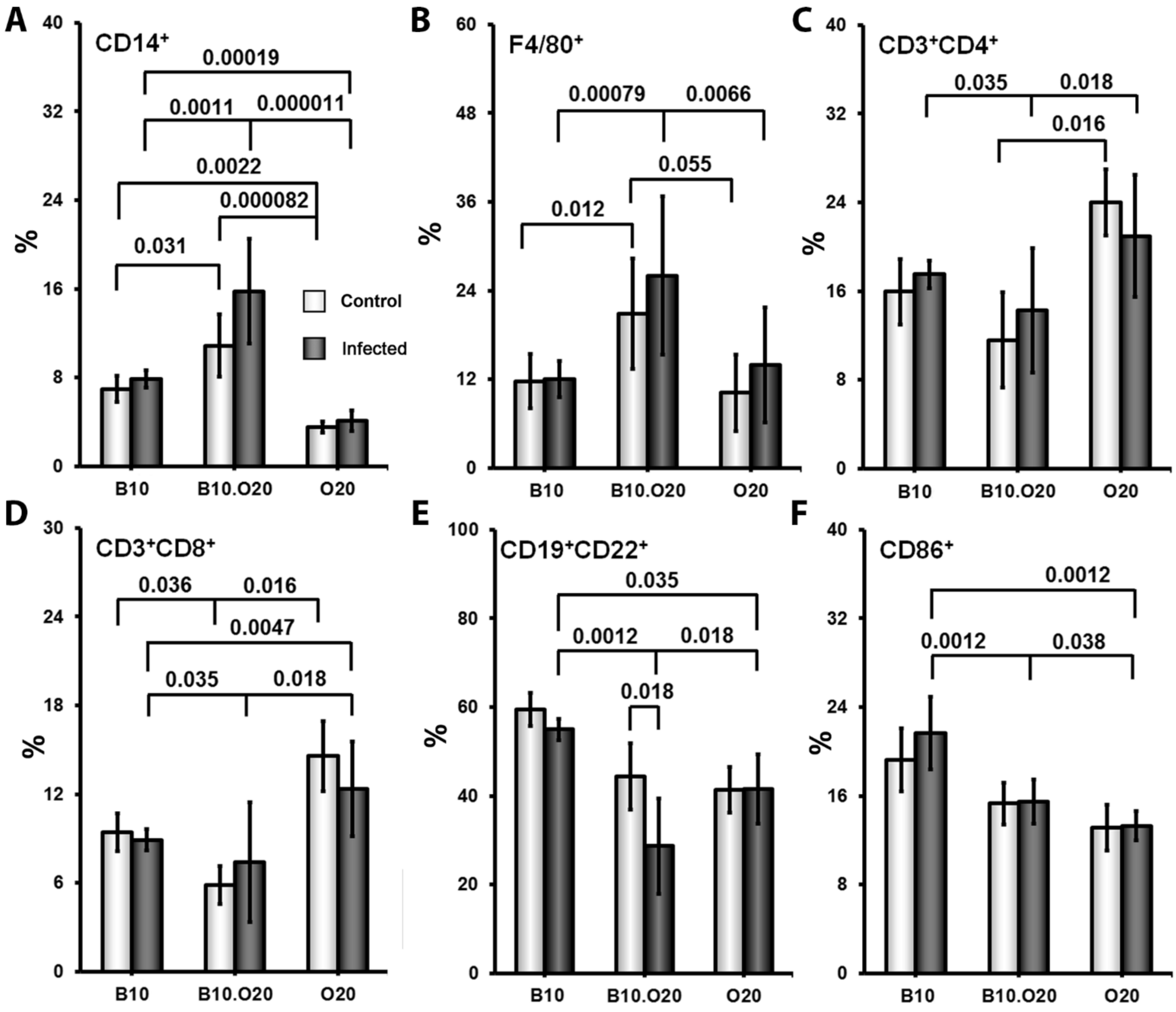

SUPPLEMENTARY FIGURE 3. Granulomas and granulomas infiltrating cells in liver of infected and uninfected mice. Female mice of the strains O20 (6 infected, 6 uninfected), B10 (6 infected, 6 uninfected) and B10.O20 (6 infected, 5 uninfected) were compared. The data show the means ± SD from two independent experiments.

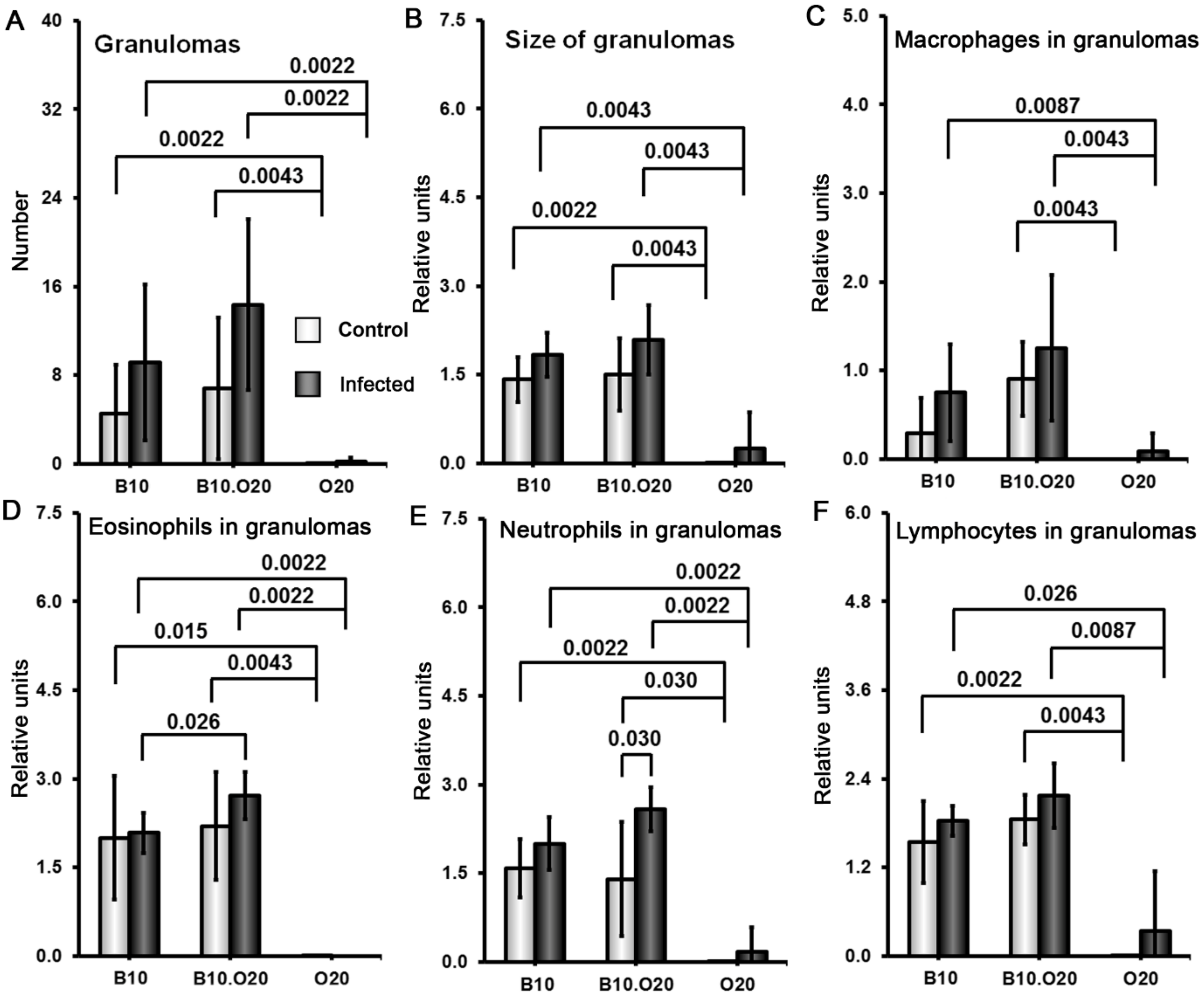

Supplement: Supplementary file 1 [file DataSheet1.pdf]
